# The Role of Genetic Polymorphisms as Related to One-Carbon Metabolism, Vitamin B6, and Gene–Nutrient Interactions in Maintaining Genomic Stability and Cell Viability in Chinese Breast Cancer Patients

**DOI:** 10.3390/ijms17071003

**Published:** 2016-06-24

**Authors:** Xiayu Wu, Weijiang Xu, Tao Zhou, Neng Cao, Juan Ni, Tianning Zou, Ziqing Liang, Xu Wang, Michael Fenech

**Affiliations:** 1 School of Life Sciences, The Engineering Research Center of Sustainable Development and Utilization of Biomass Energy, Ministry of Education, Yunnan Normal University, Kunming 650500, Yunnan, China; xiayu.wu@csiro.au or xiayu98wu@163.com (X.W.); sn-xwj@163.com (W.X.); kmzl@163.com (T.Z.); wy2057729@163.com (N.C.); gt_gg30@163.com (J.N.); liang_zq229@hotmail.com (Z.L.); 2 Third Affiliated Hospital of Kunming Medical College, Kunming 650101, Yunnan, China; zoutn@aliyun.com; 3 Genome Health and Personalized Nutrition, CSIRO Food and Nutrition, P.O. Box 10041, Adelaide SA 5000, Australia

**Keywords:** FMOCM, vitamin B6, genetic polymorphisms, gene-nutrient interaction, GSACV, breast cancer

## Abstract

Folate-mediated one-carbon metabolism (FMOCM) is linked to DNA synthesis, methylation, and cell proliferation. Vitamin B6 (B6) is a cofactor, and genetic polymorphisms of related key enzymes, such as serine hydroxymethyltransferase (SHMT), methionine synthase reductase (MTRR), and methionine synthase (MS), in FMOCM may govern the bioavailability of metabolites and play important roles in the maintenance of genomic stability and cell viability (GSACV). To evaluate the influences of B6, genetic polymorphisms of these enzymes, and gene–nutrient interactions on GSACV, we utilized the cytokinesis-block micronucleus assay (CBMN) and PCR-restriction fragment length polymorphism (PCR-RFLP) techniques in the lymphocytes from female breast cancer cases and controls. GSACV showed a significantly positive correlation with B6 concentration, and 48 nmol/L of B6 was the most suitable concentration for maintaining GSACV in vitro. The GSACV indexes showed significantly different sensitivity to B6 deficiency between cases and controls; the B6 effect on the GSACV variance contribution of each index was significantly higher than that of genetic polymorphisms and the sample state (tumor state). *SHMT C1420T* mutations may reduce breast cancer susceptibility, whereas *MTRR A66G* and *MS A2756G* mutations may increase breast cancer susceptibility. The role of *SHMT*, *MS*, and *MTRR* genotype polymorphisms in GSACV is reduced compared with that of B6. The results appear to suggest that the long-term lack of B6 under these conditions may increase genetic damage and cell injury and that individuals with various genotypes have different sensitivities to B6 deficiency. FMOCM metabolic enzyme gene polymorphism may be related to breast cancer susceptibility to a certain extent due to the effect of other factors such as stress, hormones, cancer therapies, psychological conditions, and diet. Adequate B6 intake may be good for maintaining genome health and preventing breast cancer.

## 1. Introduction

Folate, vitamin B6 (B6), and vitamin B12 (B12) have been proven to play important roles in the one-carbon metabolism pathway and the prevention of carcinogenesis: (a) in the transformation of homocysteine (Hcy) to methionine for methylation of DNA to ensure gene expression and genomic stability; and (b) in the synthesis of purine precursors and thymidylate of nucleic acid (DNA and RNA) [1,2,3,4]. B6 is not only a crucial coenzyme for serine hydroxymethyltransferase (SHMT) in the reversible transformation of serine and tetrahydrofolate (THF) to 5,10-methylene THF and glycine [5,6], a reaction that offers one-carbon units for S-adenosylmethionine (SAM), and for the synthesis of purine and pyrimidine, but it is also a cofactor for cystathionine β-synthase (CBS), which is involved in the transsulfuration pathway where Hcy is converted into cystathionine. Thus, inadequate dietary intake of B6 or deficiency of B6 in plasma might result in a state of missing DNA precursors, aberrations in DNA metabolism and histone methylation, interruption in DNA repair, and imbalance in the synthesis and degradation of Hcy, any of which are likely to be associated with promoting the development of several adverse health effects including carcinogenesis. In addition to its functions in DNA synthesis, methylation, and repair, B6 is also essential for the synthesis of glutathione (GSH) from Hcy via cysteine and cystathionine. GSH plays a key role of detoxification and protection as a cofactor of GSH peroxidases and GSH S-transferases. Additionally, it can protect cells from the oxidative damage of nucleic acids, proteins and lipids [7,8,9]. Rodent studies have shown that azoxymethane-induced colon tumorigenesis in mice is suppressed by moderate doses of dietary B6, and B6 can suppress endothelial cell angiogenesis and proliferation in part by restraining DNA topoisomerases and DNA polymerase [10,11].

Not only the above-mentioned B6 and SHMT are important in the folate-mediated one-carbon metabolism (FMOCM), which also involves various other enzymes. Among them, 5,10-methylenetetrahydrofolate reductase (MTHFR) irreversibly converts 5,10-methylene THF to 5-methyl THF [12,13]. Methionine synthase (MS) maintains methionine dynamic equilibrium by converting Hcy to methionine (the precursor of SAM) in a cobalamin-dependent reaction, which uses 5-methyl THF as the methyl donor [14]. Methionine synthase reductase (MTRR) is responsible for activating MS by keeping adequate levels of activated B12 [15,16,17,18]. The folate metabolism has attracted major attention in relation to breast carcinogenesis. In spite of its significance in the one-carbon metabolism and the elimination of oxidative stress, there is limited basic experimental proof of an association of B6 and the above-mentioned enzymes with susceptibility to cancer, especially breast cancer [19,20,21]. We previously reported associations among two polymorphisms in the MTHFR gene (677 and 1298 loci), folate (or folic acid), B12 deficiency, genome instability, and increased cell death with breast cancer risk in the Chinese Yunnan population [14,22,23,24]. Based on previous work, we utilized the cytokinesis-block micronucleus assay (CBMN) and PCR-restriction fragment length polymorphism (PCR-RFLP) techniques to determine the relationships between B6 deficiency, the other above-mentioned genetic polymorphisms in the folate pathway, gene-nutrient interactions and genomic stability and cell viability (GSACV) in vitro.

## 2. Results

### 2.1. Dose-Response Relationship between the Concentration of B6 and the Number of Viable Cells (NVC) in Cell Lines

Our goal was to ascertain the dose-response relationship between the concentration of B6 and the NVC. In preliminary experiments, we selected the lowest B6 concentrations to be 0 nmol/L with an experimental concentration gradient of 6, 12, 24, 48, 96, 200, and 4800 nmol/L to culture a breast cancer cell line and a normal cell line (GM13705 and GM12593, respectively) according to the physiological concentrations of B6 in plasma (20–40 nmol/L) [25]. Although vitamin B6 concentration in vivo is unlikely to be zero, we wanted to make sure of the growth status of the cell line in culture without B6, so we selected the lowest concentration to be 0 nmol/L. The results showed a clear dose-response growth relationship in both cell lines at various B6 concentrations, with 24 nmol/L being the lowest concentration of B6 that can lead to an increase in NVC. Because there was no significant difference of growth between 200 and 4800 nmol/L and both cell lines were dead at 0 nmol/L, we selected 6, 12, 24, 48, 96, and 200 nmol/L B6 to culture both lymphocytes from the breast cancer cases and controls in follow-up experiments (Supplementary Figure S1).

### 2.2. Comparison of GSACV with Different B6 Concentrations in the Breast Cancer Cases or Controls

The levels of GSACV biomarkers in media with different concentrations of B6 are reported in Table 1. A one-way analysis of variance (ANOVA) showed that there were no significant differences among the nuclear division index (NDI) values at different concentrations of B6 in both groups (breast cancer and controls). However, the other GSACV indexes (frequency of micronucleated binucleated cells, MNBN; micronucleated mononucleated cells, MONO; nucleoplasmic bridge cells, NPB; nuclear bud cells, NBUD) showed a decreasing trends with increasing B6 concentrations from 6–24 to 48–200 nmol/L. There were no significant differences in GSACV indexes including MNBN, NPB, NBUD, and MONO frequencies from 48 to 200 nmol/L in the same groups (breast cancer cases or controls). The frequencies of apoptosis (APO) and necrosis (NEC) significantly decreased when the concentration of B6 was raised from 6–12 to 24–200 nmol/L, and there was no significant difference in APO or NEC from 24 to 200 nmol/L B6.

A correlation analysis among the biomarkers showed that the GSACV indexes were obviously positively related to each other (*p* < 0.001–0.05, respectively) and that the GSACV indexes were significantly negatively correlated with the B6 concentration: r = −0.764, −0.615, −0.563, −0.448, −0.761, and −0.601 for MNBN, NPB, NBUD, MONO, APO and NEC (*p* < 0.001–0.05), respectively, in the breast cancer cases, and r = −0.582, −0.609, −0.574, −0.411, −0.663, and −0.510 for MNBN, NPB, NBUD, MONO, APO and NEC (*p* < 0.001–0.05), respectively, in the controls. These results are shown in Table 2 and Table 3.

### 2.3. Comparison of GSACV at the Same B6 Concentration between the Breast Cancer Cases and Controls

**Figure 1:**
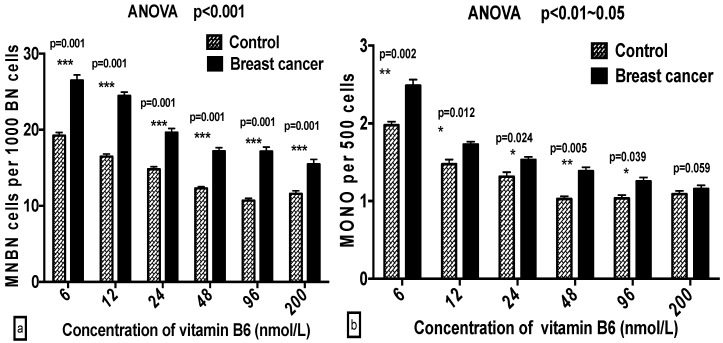

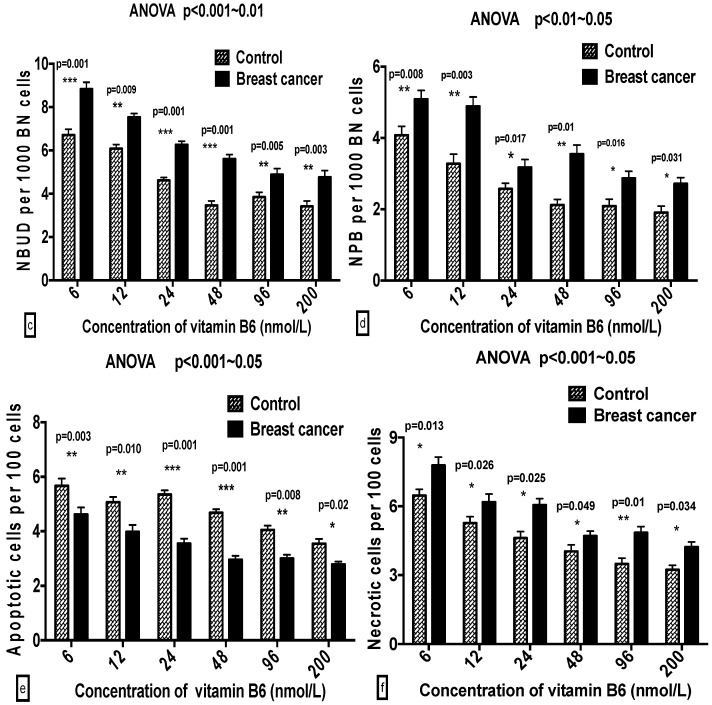
A comparison of: MNBN (**a**); MONO (**b**); NBUD (**c**); NPB (**d**); APO (**e**); and NEC (**f**) frequency between breast cancer cases and controls under various concentrations of B6.

Comparing GSACV between the breast cancer cases and controls, the MNBN, NPB, NBUD, MONO, and NEC frequencies in the controls were notably than those in the breast cancer cases at the same B6 concentrations (*p* < 0.001–0.05) (Figure 1).

### 2.4. Effect of B6 and Breast Cancer Status on GSACV

A two-way ANOVA revealed that B6 concentrations accounted for 46.15%, 30.13%, 29.66%, 3.14%, 31.65%, and 41.15% (*p* < 0.001–0.01) of the variance of the MNBN, NPB, NBUD, MONO, APO, and NEC frequencies, respectively, beside the breast cancer status, which accounted for 5.12%, 4.14%, and 5.45% of the variance of the MNBN, NPB, and NEC frequencies, respectively (*p* < 0.05). The investigation suggested that the breast cancer status affected GSACV but that the effect was less significant than that of the B6 concentration (Table 4).

**Figure 2:**
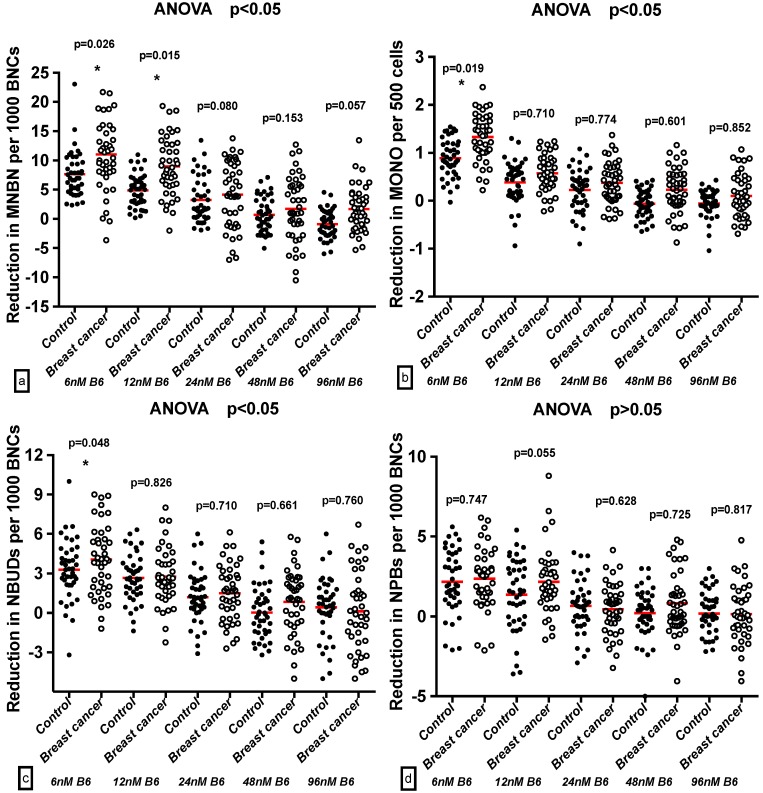

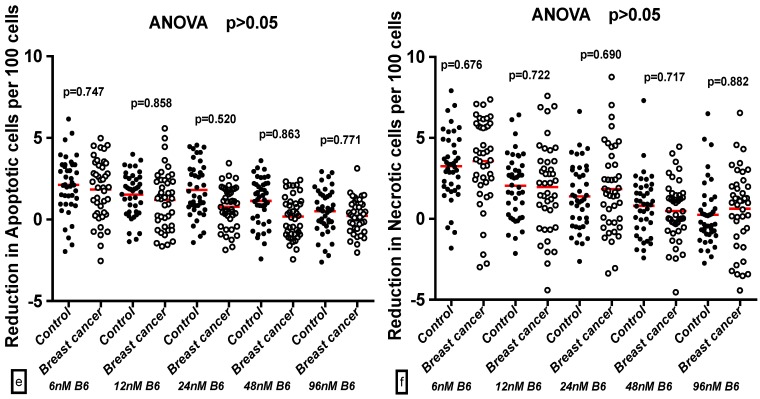
A comparison of reduction of: MNBN (**a**); MONO (**b**); NBUD (**c**); NPB (**d**); APO (**e**); and NEC (**f**) frequency between breast cancer cases and controls using DDA. The results represent the reduction in markers measured relative to 200 nmol/L mean ± SE.

The breast cancer cases and controls had distinct genomic baselines, which are derived from their different genetic backgrounds. Thus, we performed a Difference in Difference Analysis (DDA) [26] by subtracting the pooled values of the biomarker for 6 to 96 nmol/L from the scores for 200 nmol/L B6. Interestingly, we observed that the MNBN values in the breast cancer cases were significantly higher than those in the respective controls at 6 and 12 nmol/L B6 (*p* < 0.05). Meanwhile, the MONO and NBUD levels were notably higher than those in the controls at only 6 nmol/L (*p* < 0.05). There were no significant differences in the NPB, APO, and NEC occurrences between the breast cancer cases and controls. The analyses showed that there were significant differences in sensitivity to the genotoxic and cytotoxic effects of B6 deficiency between the breast cancer cases and controls with respect to MNBN, MONO, and NBUD frequencies (Figure 2).

### 2.5. The GSACV Indexes in Different SHMT C1420T, MS A2756G, and MTRR A66G Genotypes in Breast Cancer Cases and Controls at Various B6 Concentrations

**Figure 3:**
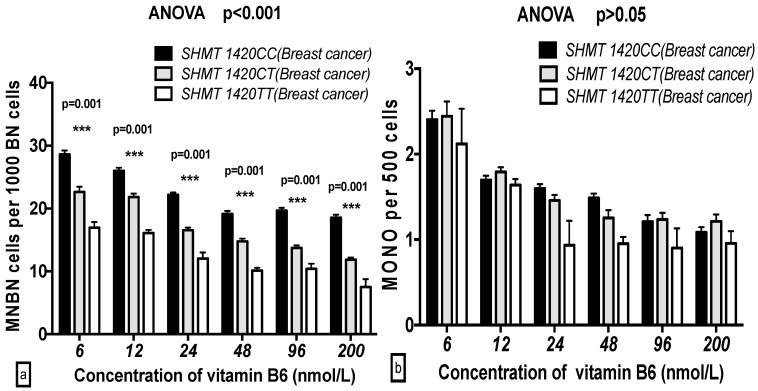

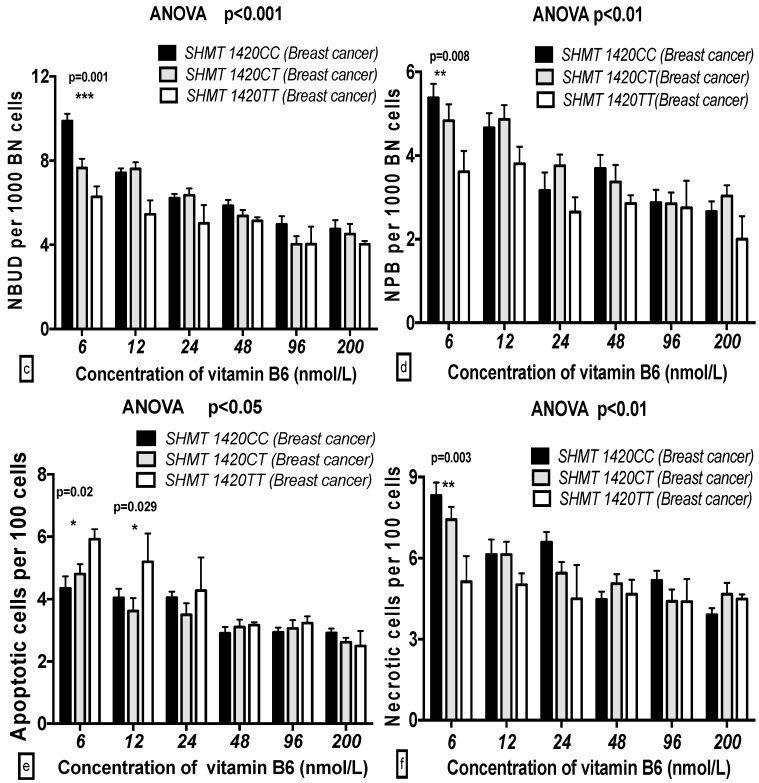
GSACV including: MNBN (**a**); MONO (**b**); NBUD (**c**); NPB (**d**); APO (**e**); and NEC (**f**) frequency in different *SHMT C1420T* genotypes of breast cancer cases at various concentrations of B6 (*CC* = 24, *CT* = 16, *TT* = 2 in breast cancer cases).

**Figure 4:**
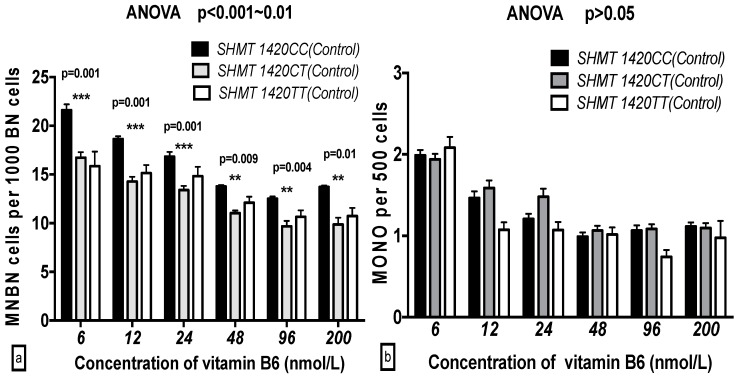

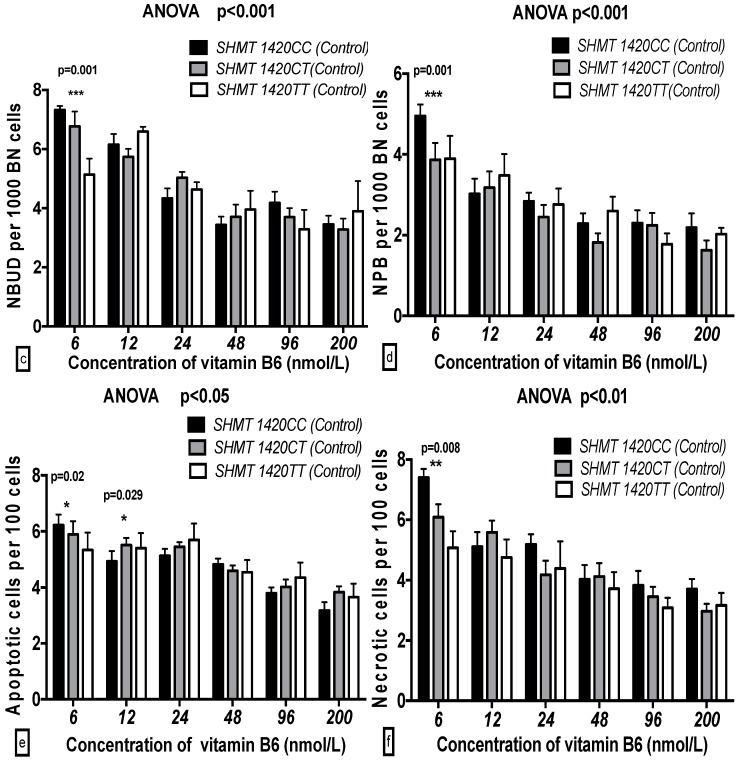
GSACV including: MNBN (**a**); MONO (**b**); NBUD (**c**); NPB (**d**); APO (**e**); and NEC (**f**) frequency in different *SHMT C1420T* genotypes of controls at various concentrations of B6 (*CC* = 17, *CT* = 19, *TT* = 6 in controls).

There were significant differences in the GSACV indexes (MNBN, NPB, NBUD, APO and NEC) that were observed with the different *SHMT C1420T* genotypes at the same B6 concentration in the breast cancer cases. In contrast, no significant differences in the GSACV indexes (NPB, NBUD, MONO, APO and NEC) with the exception of MNBN, were observed with the different *SHMT C1420T* genotypes at the same B6 concentration in the control groups. There was a significant increase in the MNBN frequency for the *SHMT 1420CC* vs *SHMT 1420TT* genotypes at the same concentration of B6 (*p* < 0.001) in both the breast cancer cases and controls. The NBUD, APO, and NEC frequencies of *SHMT 1420CC* were higher than those of *SHMT 1420TT* at 6 and 12 nmol/L B6, while the NPB frequency of *SHMT 1420CC* was higher than that of *SHMT 1420TT* only at 6 nmol/L B6 in the breast cancer cases (Figure 3 and Figure 4).

**Figure 5:**
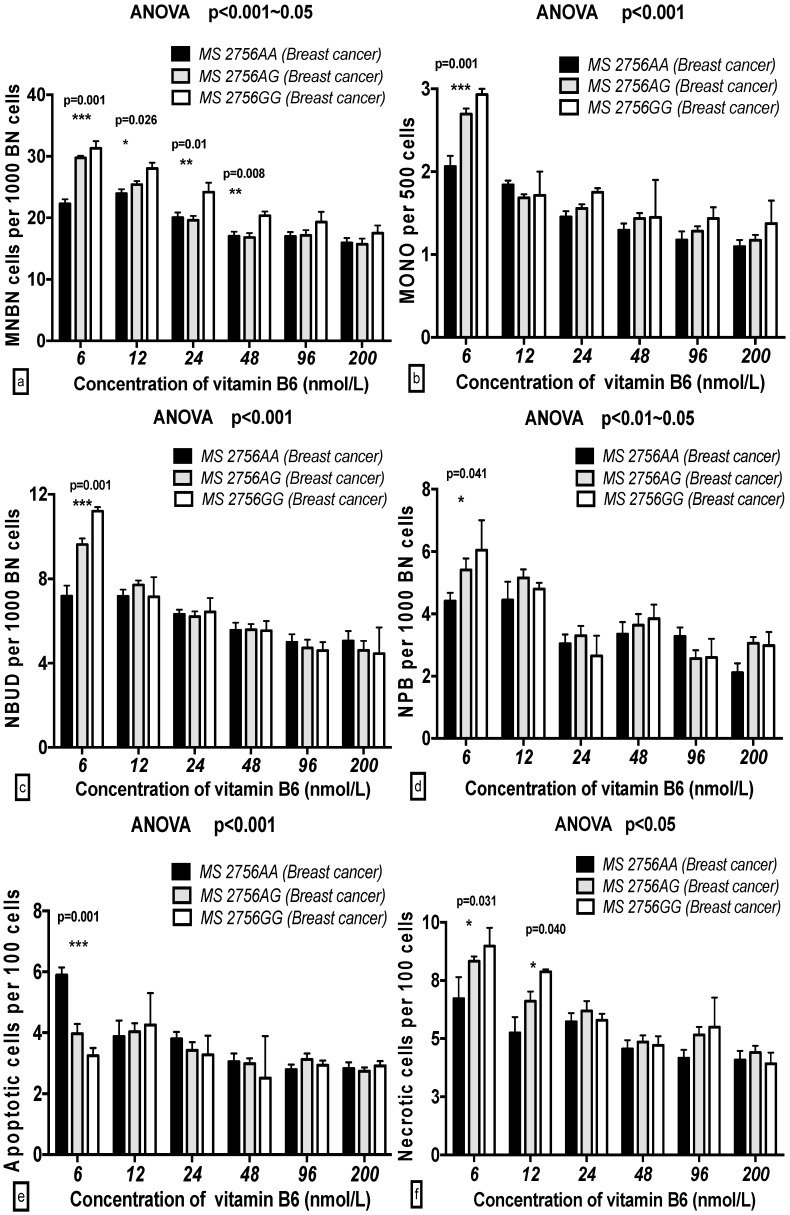
GSACV including: MNBN (**a**); MONO (**b**); NBUD (**c**); NPB (**d**); APO (**e**); and NEC (**f**) frequency in different *MS A2756G* genotypes of breast cancer cases at various B6 concentrations (*AA* = 15, *AG* = 25, *GG* = 2 in breast cancer cases).

**Figure 6:**
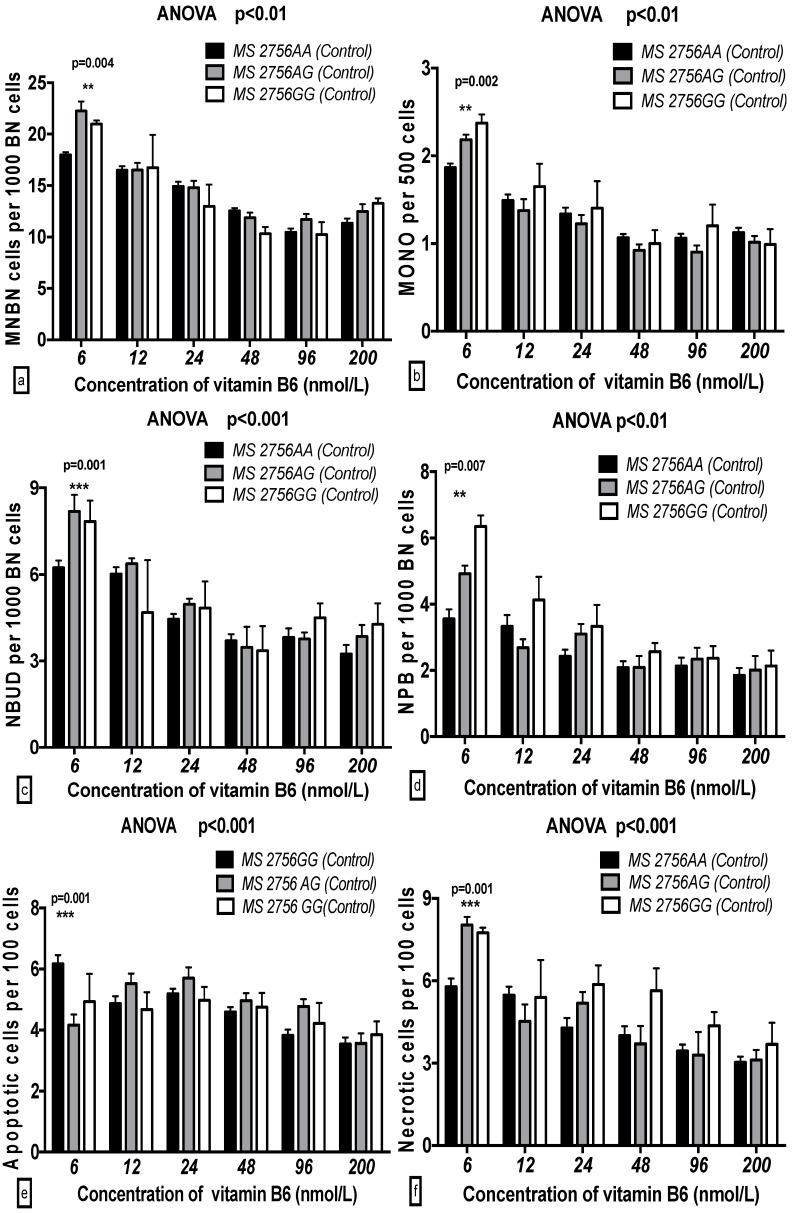
GSACV including: MNBN (**a**); MONO (**b**); NBUD (**c**); NPB (**d**); APO (**e**); and NEC (**f**) frequency in different *MS A2756G* genotypes of controls at various B6 concentrations (*AA* = 29, *AG* = 10, *GG* = 3 in controls).

In the breast cancer cases, the MNBN frequency with the *MS 2756GG* genotype was significantly increased compared to that with the *MS 2756AA* genotype at 6 to 48 nmol/L B6 (*p* < 0.001–0.05), whereas the MONO, NBUD, NPB, and NEC frequencies with the *MS 2756GG* genotype were significantly enhanced compared to those with the *MS 2756AA* genotype at 6 and 12 nmol/L B6 (*p* < 0.001–0.05). The APO frequency with the *MS 2756GG* genotype was enhanced at 6 nmol/L B6. In the controls, none of the GSACV (MNBN, MONO, NPB, NBUD, APO and NEC) indexes were significantly different for the different genotypes at the same concentration of B6 (Figure 5 and Figure 6).

**Figure 7:**
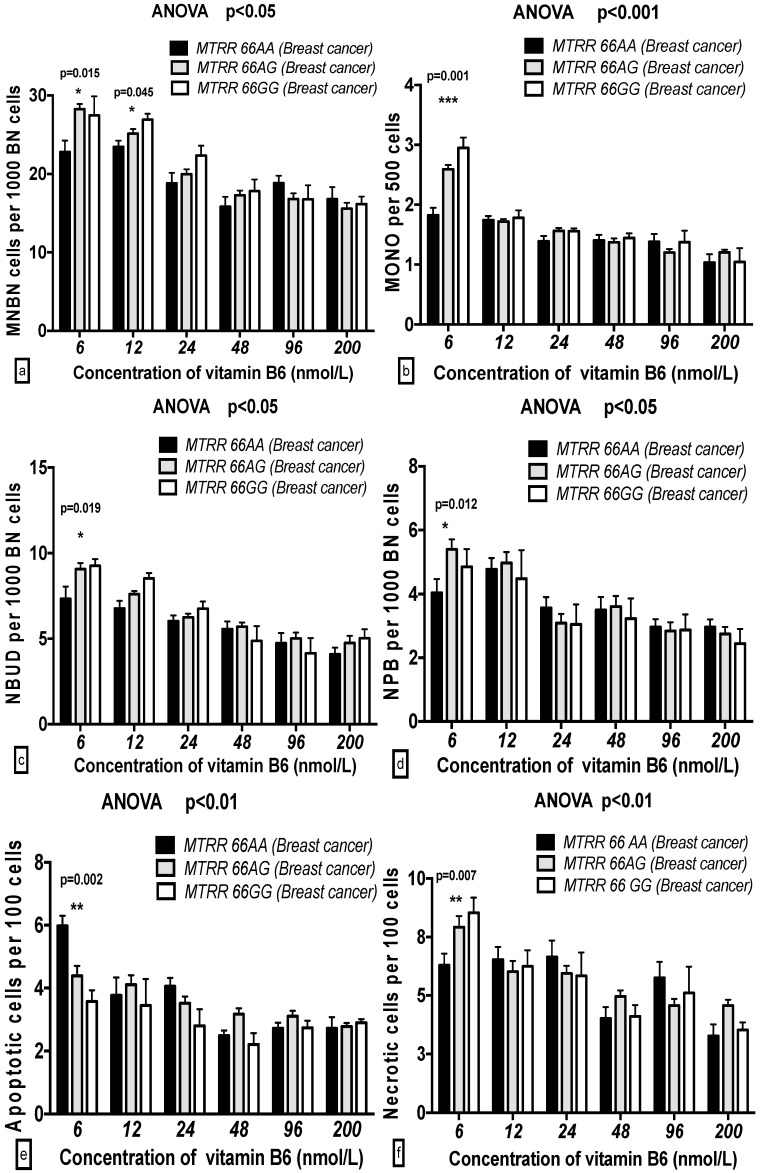
GSACV including: MNBN (**a**); MONO (**b**); NBUD (**c**); NPB (**d**); APO (**e**); and NEC (**f**) frequency in different *MTRR A66G* genotypes of breast cancer cases at various B6 concentrations (*AA* = 8, *AG* = 30, *GG* = 4 in breast cancer cases).

**Figure 8:**
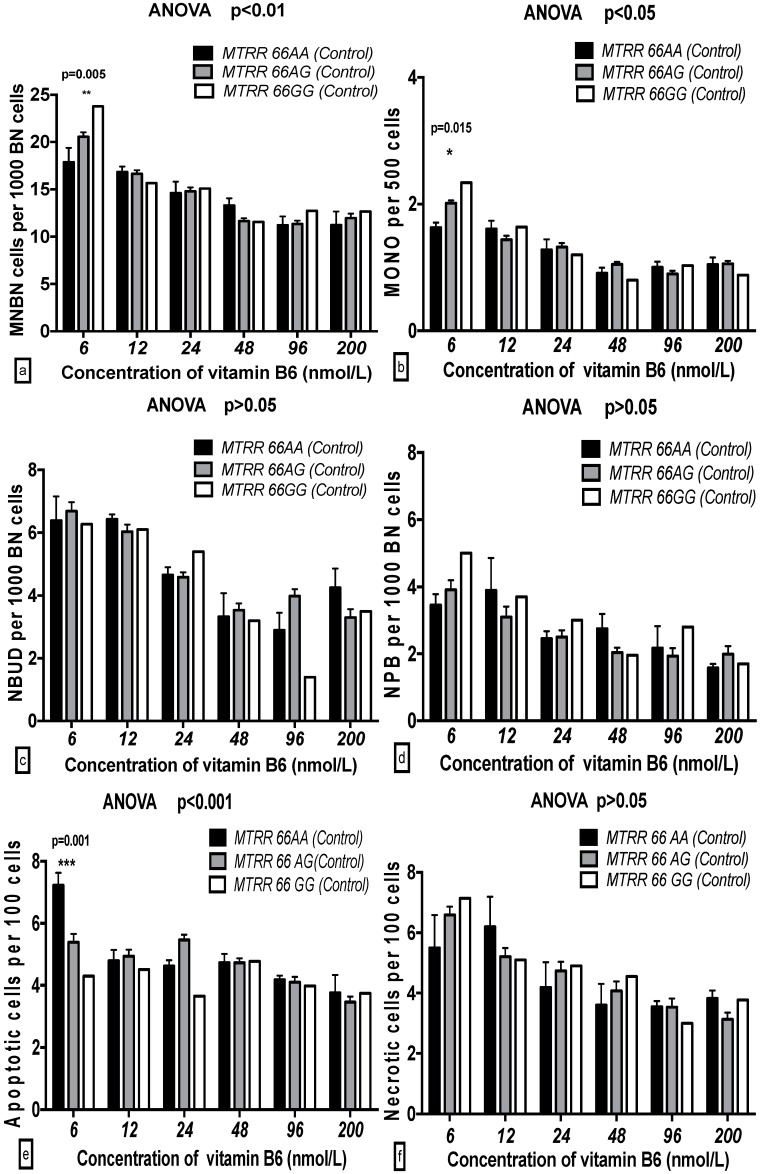
GSACV including: MNBN (**a**); MONO (**b**); NBUD (**c**); NPB (**d**); APO (**e**); and NEC (**f**) frequency in different *MTRR A66G* genotypes of controls at various B6 concentrations (*AA* = 5, *AG* = 36, *GG* = 1 in controls).

The MNBN, MONO, and APO frequencies with the *MTRR 66GG* genotype were significantly increased compared to those with the *MTRR 66AA* genotype at 6 nmol/L B6 in both the breast cancer cases and controls. The NBUD frequency with the *MTRR 66AA* genotype was markedly decreased compared to that of *MTRR 66GG* at 6 nmol/L B6 only in the breast cancer cases and not in the controls (Figure 7 and Figure 8).

### 2.6. The Effects of the Strength and Variation Analysis of B6 and SHMT C1420T, MS A2756G, and MTRR A66G Polymorphisms on GSACV by Two-Way ANOVA

A two-way ANOVA indicated that the B6 concentration accounted for 36.07%, 20.81%, 30.42%, 41.87%, 19.88%, and 22.73% of the variance of the MNBN, NPB, NBUD, MONO, NEC, and APO frequencies, respectively (*p* < 0.001). However, the *SHMT C1420T* polymorphisms only explained 21.10%, 2.18%, and 2.31%, of the MNBN, MONO, and NEC frequencies (*p* < 0.001–0.01), respectively, in the controls. The B6 concentration accounted for 16.58%, 7.19%, 11.94%, 21.60%, 5.68%, and 13.09% of the variance of the MNBN, NPB, NBUD, MONO, NEC, and APO frequencies, respectively (*p* < 0.001). However, the *SHMT C1420T* polymorphisms only explained 30.36%, 1.56%, and 3.21% of the MNBN, MONO, and NBUD frequencies (*p* < 0.001–0.01), respectively, in the breast cancer cases. There was only an interaction between the B6 concentration and the *SHMT C1420T* polymorphisms for the NBUD biomarker in the breast cancer cases (Table 5).

The B6 concentration accounted for 28.45%, 20.23%, 17.77%, 35.30%, 18.19%, and 7.98% of the variance of the MNBN, NPB, NBUD, MONO, NEC, and APO frequencies, respectively (*p* < 0.001). However, the *MS A2756G* polymorphisms only explained 1.31%, 1.45%, and 2.76% of the MNBN, NBUD, and NPB frequencies (*p* < 0.001–0.01), respectively, in the controls. The B6 concentration accounted for 20.68%, 11.68%, 19.86%, 23.69%, 12.71%, and 6.98% of the variance of the MNBN, NPB, NBUD, MONO, NEC, and APO frequencies, respectively (*p* < 0.001). However, the *MS A2756G* polymorphisms only explained 2.89%, 2.23%, and 3.57% of the MNBN, MONO, and NEC frequencies (*p* < 0.001–0.01), respectively, in the breast cancer cases. There was an interaction between the B6 concentration and the *MS A2756G* polymorphisms for the MNBN, MONO, APO, and NEC biomarkers in the breast cancer cases and for the MNBN, MONO, NBUD, and APO biomarkers in the controls (Table 6).

The B6 concentration accounted for 13.79%, 17.03%, 10.03%, 11.74%, 5.09%, and 3.73% of the variance of the MNBN, NPB, NBUD, MONO, NEC, and APO frequencies, respectively (*p* < 0.001). However, there were no effects on the biomarkers for the *MTRR A66G* polymorphisms in the controls. The B6 concentration explained 26.69%, 11.95%, 25.31%, 32.47%, 17.72%, and 13.95% of the variance of the MNBN, NPB, NBUD, MONO, NEC, and APO frequencies, respectively (*p* < 0.001). However, the *MTRR A66G* polymorphisms only explained 1.40% and 1.44% of the MONO and NBUD frequencies (*p* < 0.01–0.05), respectively, in the breast cancer cases. There was only an interaction between the B6 concentration and the *MTRR A66G* polymorphisms for the NBUD biomarker in the breast cancer cases (Table 7).

## 3. Discussion

In the present study, we evaluated the response of the lymphocytes from women with breast cancer to B6 deficiency and gene-nutrient interactions by analyzing the induction of genomic damage and the effect of B6 on cell proliferation and viability. A deficiency of B6 increased genomic instability in binucleated cells. This was an expected result because folate and B6 have been shown to be connected to breast carcinogenesis [27,28]. B6, as a critical coenzyme, acts in two different steps of the folate metabolism pathway: one is the synthesis of 5,10-methylenetetrahydrofolate, which is crucial for DNA synthesis, DNA repair, and DNA methylation [29,30], and the other is the Hcy to glutathione catabolism, which plays vital roles in the detoxification as well as in the defense of cells from oxidative DNA damage [31]. Secondly, the results showed that cell viability increased at 24 nmol/L B6, indicating that proper B6 nutrition is a key determinant of cell growth and proliferation. The CBMN analysis is a thorough system for evaluating genomic damage, cytotoxicity, and cytostasis. Genomic damage events are marked especially in once-divided binucleated (BN) cells, which include three indexes: (a) the chromosome breakage and/or whole chromosome loss biomarker of micronuclei (MNi); (b) the DNA misrepair and/or telomere end-fusions biomarker of NPBs; and (c) the elimination of amplified DNA and/or DNA repair complexes biomarker of NBUDs. The cytostatic events are scored, including frequencies of mononucleated cells, binucleated cells, and multinucleated cells, and cytotoxic events are counted via necrotic and apoptotic cell proportions.

### 3.1. Influences of B6 on GSACV

We hypothesized that decline in the MNBN frequencies with increasing B6 concentration may be due to a slowdown of the cell proliferation speed or to the wear of strongly damaged cells [22,23,24]. Anyhow, the cells will not arrive at the mitotic telophase when MNBNs are marked in BN cells. The responses to DNA damage are serious and can decrease cell viability, and they can further induce the activation of surveillance systems, which either postpones cell cycle progression until the damaged DNA is repaired or eliminated, or induces apoptosis in case the damage is irreparable [32]. Additionally, we inspected whether the B6 deficiency-induced MNBNs from 9 day would be accompanied by a reduction in NVC and the NDI of the breast cancer cases and controls. Interestingly, our results showed that there was no difference in NDI among the different concentrations of B6. B6 deficiency cultures showed a regular progression through the cell cycle, which maintained the cells’ proliferation ability without suffering either cell loss or the prospective mitotic delay regardless of the persistent DNA lesions [33,34].

We separately analyzed the pooled data from this study and verified that the B6 concentration correlated significantly negative with all of these markers of DNA damage and cell death. DNA damage was minimized at 48 to 200 nmol/L B6 in the breast cancer cases and controls; these concentrations are greater than those normally observed in plasma (20–40 nmol/L) [25]. Cell death was minimized at 24 to 200 nmol/L B6, which suggested that the B6 concentration in plasma may only maintain cell viability, but is not sufficient for maintaining genomic stability. We conclude that 48 nmol/L B6 is the optimal concentration for cell viability and genome stabilization in vitro. More interestingly, we observed that the MNBN, NPB, NBUD, and NEC frequencies correlated significantly and positively, suggesting that whole chromosomes or fragments that lag behind at anaphase may be related to increased chromosome rearrangement and gene amplification [35].

The notion that genetic susceptibility to carcinogenesis is connected to genomic instability was originally observed in rare disorders such as xeroderma pigmentosum and ataxia telangiectasia, which are associated with in vitro and in vivo chromosomal instability and defective DNA repair capacity [36]. The matched-pair Student’s *t*-test revealed that the breast cancer status in the cases contributed to the variations in the measured biomarkers. The MNBN, NPB, NBUD, MONO, APO, and NEC frequencies in the cancer cases were markedly modified by the B6 concentrations as compared to the controls. This result suggests that breast cancer cases are more sensitive to B6 deficiency than matched controls, considering genomic instability and cell death, which stresses the possibility that B6 may be more important to genome maintenance in cancer groups than in healthy groups. As there have been no studies to date on the relationship between B6 and genomic stability in breast cells, we cannot exclude the possibility that the sensitivities of lymphocyte cells and breast epithelium to B6 deficiency may be different and that the estrogen level in different individuals may have influenced our results. Therefore, future research on the effects of micronutrient deficiency on GSACV should be considered in breast cells, lymphocytes, and tumor patients by including different cancer cells, types of cells, and hormone expression levels.

### 3.2. Influences of Genetic Polymorphisms on GSACV

We examined the association between GSACV and three enzyme polymorphisms, *SHMT C1420T*, *MS A2756G*, and *MTRR A66G*, under B6 deficiency, which was based on functional polymorphisms and previous reports of associations with breast cancer risk. Functional polymorphisms in these genes may alter the availability of folate in the synthesis and methylation of DNA, and may consequently influence the susceptibility to cancer. To our knowledge, this is also the first report examining the association between *MTHFR C677T* polymorphisms and GSACV in a Chinese population, though the data are limited for most other populations [37].

In our study, we found an association between the individual *SHMT C1420T* polymorphism studied and genomic stability. *SHMT 1420TT* genotypes were found to have reduced DNA damage in the breast cancer cases and controls as compared to *CC* genotype. One key reason is that the variant SHMT enzyme may lead to accumulation of THF and reduced production of 5,10-methylene THF, although the exact biological effect of this phenomenon or the complete mechanism leading to DNA damage is not certain. Our previous study underlines the capacity of the *SHMT C1420T* polymorphism being able to directly reduce breast cancer risk. [14,38]. The study found risk reduction for the *SHMT 1420TT* genotype in malignant lymphomas [39], adult acute lymphocytic leukemia [40], and also in a wide spectrum of diseases including Parkinson’s disease (PD), coronary artery disease (CAD) and systemic lupus erythematosus (SLE) [38]. These suggested that the reduction of DNA damage in the *SHMT 1420TT* genotype might be the main reason for the risk reduction of different cancers and a wide spectrum of diseases. The variant SHMT enzyme may result in decreased production of 5,10-methylene THF and decreased accumulation of THF, even though the accurate and complete biology mechanism leading to DNA damage is not recognized.

For the *MS 2756AG* polymorphism, increased GSCAV was found for the *AA* genotype as compared to the *GG* genotype. For *MTRR A66G*, the results indicated elevated GSACV for carriers of the homozygote wild-type genotype (*AA*) as compared to the other genotypes. The main reason for increased GSCAV is that the *A2756G* mutation in the *MS* gene leads to the Asp919Gly substitution in the MS enzyme, which further results in more valid production of methionine and remethylation and the elevation of Hcy, as well as DNA hypomethylation [41]. For *MTRR A66G*, it was revealed that the *66GG* genotype was inversely related to Hcy levels in plasma. Additionally, the mutation of *MTRR 66AG* easily showed the irregular methylation of DNA and abnormal nucleotide synthesis and altered repair. Our previous study showed that *MS 2756GG* and *MTRR 66GG* genotypes had significantly increased risk among Chinese women, which might be due to higher GSCAV.

### 3.3. Influences of Gene-Nutrient Interactions on GSACV

The interaction between *SHMT C1420T*, *MS A2756G*, and *MTRR A66G* polymorphisms and B6 deficiency was addressed in vitro with human lymphocytes cultured at different concentrations of B6. The different modifying effect of the *SHMT C1420T*, *MS A2756G*, and *MTRR A66G* polymorphisms on various cytogenetic markers may indicate that under B6 deprivation, MNi and NPBs arise through different mechanisms. The results suggest that B6, *SHMT C1420T*, and *MS A2756G* are three of the basic determining factors of lymphocyte growth in culture medium, while *MTRR A66G* has less effect on it. In addition, it is evident that the *SHMT 1420TT* and *MS 2756AA* genotypes provided a notable growth advantage over the *SHMT 1420CC* and *MS 2756GG* genotypes. The growth advantage of the *SHMT 1420TT*, *MS 2756AA*, and *MTRR 66AA* genotypes may be concerned with decreased cell cycle delay, which may result from the NBUDs formation process which happens during S-phase [42,43,44]. The cells bearing *SHMT 1420TT*, *MS 2756AA*, and *MTRR 66AA* express significantly fewer NBUDs than *SHMT 1420CC*, *MS 2756GG*, and *MTRR 66GG* cells. An alternative interpretation is the slight reduction in the APO frequency in *SHMT 1420TT*, *MS 2756AA*, and *MTRR 66AA* cells compared with *SHMT 1420CC*, *MS 2756GG*, and *MTRR 66GG* cells. Lee et al. examined the relationship between polymorphisms of *MTHFR A1298C*, and *C677T*, *MTRR A66G* and *MTR A2756G* and non-obstructive male infertility in a Korean population. They found that *MTHFR C677T*, *MTRR A66G*, and *MS A2756G* genotypes were independently related to male infertility [45]. Ethnic differences, gender, geographic variation, the distribution of folate-related enzyme gene polymorphisms, as well as gene-nutrient, gene-environmental, and gene-ethnic interactions have been shown to influence the impact of GSACV.

There were limitations in the present study: (a) the sample number in this study is relatively small, especially the *MTRR 66GG* genotype of the controls which has only one subject, which could decrease the statistical credibility of finding divergence between breast cancer cases and controls; and (b) all samples were selected from one hospital and all samples were limited to females, which could not explain populations in other places and males. Therefore, a larger number of samples and geographic variation studies are greatly needed for studies in the future.

## 4. Materials and Methods

### 4.1. Cell Lines

The GM12593 and GM13705 cell lines are both human B lymphoblastoid cell lines. Both cell lines are from NIGMS Human Genetic Mutant Cell Bank (NIGMS Human Genetic Mutant Cell Repository), and were supplied by Prof. Michael Fenech, Adelaide, Australia. GM12593 came from the spleen of a 34-year-old Caucasian female, while GM13705 was derived from a 38-year-old Caucasian female carrying a positive family history of breast cancer and with a germline *BRCA1* mutation at exon 11, codon 1252. The mutation results in the production of a truncated protein [46].

### 4.2. Characteristics of the Study Population

Consent for our study was acquired from the Yunnan Scientific and Technological Committee and the National Natural Sciences Foundation of China (NSFC). All breast cancer cases and controls agreed to participate and provided written informed consent. Pathology tests were fulfilled, and a number of active manifestations were recruited from each patient. The breast cancer cases were randomly picked from January 2010 to April 2011 in the Third Affiliated Hospital of Kunming Medical College, Kunming, Yunnan, China. In view of the hospital chart number, the breast cancer cases included 42 females consecutively selected from subjects with a first confirmed histopathologic breast carcinoma diagnosis in the age range of 49.83 ± 13.6 years, and 42 female controls comprising individuals without a history of cancer with ages in the range of 43.15 ± 12.6 years were simultaneously recruited from the health examination clinics in the same hospital during the same study period. These samples are the same as in our previous study [14].

### 4.3. Genotyping Analysis

Peripheral blood lymphocyte*s* were collected from each subject for CBMN assay. Genomic DNA was extracted from fresh or frozen whole blood using a commercially available FlexiGen DNA isolation kit (Qiagen, Valencia, CA, USA). Characteristics of the studied polymorphisms of *SHMT C1420T*, *MS A2756G*, and *MTRR A66G* are reported in our previous study [14]. All genotypes were determined by PCR-based assays followed by RFLP analysis according to published methods [14,47,48,49]. In addition, for internal quality control, 90% of samples were repeated. The genotypes of *SHMT 1420CC*/*MTRR 66AA*/*MS 2756AA* were defined as wild homozygous, *SHMT 1420CT*/*MTRR 66AG*/*MS 2756AG* as mutant heterozygous, and *SHMT 1420TT*/*MTRR 66GG*/*MS 2756GG* as mutant homozygous.

### 4.4. Cell Lines and Lymphocytes Culture

Before culturing, GM13705 and GM12593 cell lines were washed with fresh Hank’s balanced salt solution (HBSS, Biochrom AG, Berlin, Germany) three times. Cells were prepared at a concentration of 0.25 × 10^6^ viable cells/mL for 2 mL volumes in Roswell Park Memorial Institute Medium (RPMI) 1640 culture medium containing doses of B6 (0, 6, 12, 24, 48, 96, and 200 nmol/L). B6 in regular RPMI 1640 medium (4800 nmol/L) was used as a control. The lymphocytes were cultured at 0.5 × 10^6^ viable cells/mL in 2 mL volume in RPMI 1640 culture medium containing different doses of B6 (6, 12, 24, 48, 96, and 200 nmol/L). Mitogenesis was stimulated by the addition of phytohaemagglutinin (45 g/mL) (PHA; Murex Biotech, Kent, UK) and cultures were incubated at 37 °C and 5% CO_2_ in a humidified incubator. After 3 days, cell number and viability were determined using a Coulter counter and Trypan blue exclusion, respectively. The cultures were continued in 4.7 mL fresh medium and 0.3 mL “conditioned” medium from the previous 3-day culture with 0.5 × 10^6^ viable cells/mL. The components of fresh medium were the same as above but without PHA. Medium change was repeated at 6 days post-PHA treatment and a final viable cell count was measured on day 9. On the eighth day post-PHA treatment, two 750 μL aliquots of each culture were transferred to 6 mL culture tubes for the CBMN. Cytochalasin B (4.5 g/mL; Sigma Chemical Co., Darmstadt, Germany) was added to each tube and approximately 28 h later cells were harvested onto microscope slides using a cyto-centrifuge (Shandon Southern Products, Cheshire, UK). All other constituents of the medium were standard for RPMI 1640 and CBMN assay was performed as previously described [14,50,51]. All experiments in this study were conducted in the dark to avoid light influences.

### 4.5. Statistical Analysis

The results for comparison of GSACV with different B6 concentrations in the breast cancer cases or controls were compared using a one-way ANOVA. The GSACV of two groups were compared using the matched-pair Student’s *t*-test. The significant differences between B6 and genotype were determined by a repeated-measure two-way ANOVA. The differences in sensitivity to B6 deficiency between the breast cancer and control groups were determined using a DDA [26]. The mean values of the GSACS indexes for the breast cancer cases and controls with different genotypes under various concentrations of B6 were determined using the Newman-Keuls ANOVA post-test (two-tailed). The graphical analysis and statistical analyses were conducted using the Prism 6.0 software (GraphPad, San Diego, CA, USA) and SPSS Statistical Package,Version 12.0 (IBM SPSS Inc., Chicago, IL, USA).

## 5. Conclusions

In conclusion, this research indicates that B6 deficiency induces the formation of MNBNs, NPBs, and NBUDs, decreases APO and increases NEC frequencies in vitro, which shows that it may be a risk factor for cancer by inducing genomic instability/hypermutability/gene amplification in cells via chromosome breakage/rearrangement and breakage-fusion-bridge cycles. Human genome instability can be induced by vitamin B6 deficiency, and 48 nmol/L B6 was the most suitable concentration to maintain genomic stability in lymphocytes in vitro. Although in vitro conditions might not precisely estimate internal B6 requirements, these conditions offer a valuable guideline for the optimal concentration range to maintain genome health. Breast cancer patients are more sensitive to B6 deficiency than controls with respect to genomic stability, suggesting that a low intake of B6 in the long term could increase DNA damage and further exacerbate genomic instability. Adequate B6 intake was believed to be beneficial for breast cancer prevention. In addition, this research showed that the *SHMT C1420T* mutation may reduce breast cancer susceptibility. In contrast, the *MS A2756G* and *MTRR A66G* mutations may increase breast cancer susceptibility, but the role of polymorphisms in *SHMT*, *MS*, and *MTRR* in genome stability is reduced compared to that of B6, which is consistent with our previous results [17]. These results suggest that individuals with various genotypes have different sensitivities to B6 deficiency. FMOCM metabolic enzyme gene polymorphisms may be associated with breast cancer.

## Figures and Tables

**Table 1:** The indexes of GSACV in breast cancer cases and controls under various B6 concentrations.

		B6 in Medium (nmol/L) ($\bar{x}$ ± SD) ^1,2,3^					
Endpoint	Group	6	12	24	48	96	200
NDI	Cases	1.30 ± 0.026 ^a^	1.28 ± 0.028 ^a^	1.29 ± 0.021 ^a^	1.36 ± 0.041 ^a^	1.30 ± 0.034 ^a^	1.30 ± 0.027 ^a^
	Controls	1.26 ± 0.027 ^a^	1.27 ± 0.031 ^a^	1.25 ± 0.015 ^a^	1.26 ± 0.030 ^a^	1.23 ± 0.027 ^a^	1.28 ± 0.025 ^a^
‰ MNBN	Cases	27.8 ± 2.05 ^a^	25.9 ± 1.60 ^b^	22.2 ± 3.24 ^b^	18.7 ± 2.44 ^c^	17.2 ± 1.62 ^c^	17.0 ± 2.63 ^c^
	Controls	20.4 ± 2.12 ^a^	17.4 ± 1.45 ^b^	15.7 ± 3.30 ^c^	13.6 ± 2.23 ^c,d^	12.07 ± 1.61 ^d^	13.44 ± 1.17 ^d^
‰ MONO	Cases	2.78 ± 0.45 ^a^	1.74 ± 0.40 ^b^	1.62 ± 0.65 ^b^	1.55 ± 0.53 ^b^	1.44 ± 0.23 ^b^	1.40 ± 0.42 ^b^
	Controls	2.05 ± 0.45 ^a^	1.59 ± 0.59 ^a^	1.49 ± 0.28 ^a,b^	1.30 ± 0.30 ^b^	1.29 ± 0.35 ^b^	1.33 ± 0.40 ^b^
‰ NBUD	Cases	9.30 ± 1.82 ^a^	7.98 ± 2.26 ^b^	6.13 ± 1.11 ^b,c^	5.96 ± 0.74 ^c^	5.43 ± 1.33 ^c^	6.02 ± 0.95 ^c^
	Controls	7.12 ± 1.07 ^a^	6.03 ± 1.52 ^b^	4.64 ± 1.42 ^b^	4.08 ± 1.44 ^b,c^	4.21 ± 1.07 ^b,c^	4.00 ± 0.68 ^c^
‰ NPB	Cases	5.36 ± 0.76 ^a^	4.93 ± 1.22 ^a^	3.76 ± 0.42 ^a,b^	3.41 ± 0.55 ^b^	3.08 ± 1.05 ^b^	3.11 ± 0.80 ^b^
	Controls	4.01 ± 0.27 ^a^	3.41 ± 0.56 ^a^	2.91 ± 0.67 ^b^	2.46 ± 0.60 ^b^	2.46 ± 1.02 ^b^	2.02 ± 0.12 ^b^
% APO	Cases	4.59 ± 0.80 ^a^	4.10 ± 0.67 ^a,b^	3.58 ± 1.10 ^b,c^	3.37 ± 1.11 ^b,c^	3.02 ± 0.95 ^b,c^	2.86 ± 0.97 ^c^
	Controls	5.22 ± 0.55 ^a^	5.09 ± 0.80 ^a,b^	5.71 ± 0.95 ^b,c^	5.25 ± 0.62 ^b,c^	4.24 ± 0.67 ^b,c^	3.97 ± 0.67 ^c^
% NEC	Cases	7.83 ± 0.53 ^a^	5.93 ± 0.40 ^b^	5.89 ± 0.60 ^b,c^	5.02 ± 0.78 ^c^	5.65 ± 0.63 ^c^	4.73 ± 0.73 ^c^
	Controls	6.37 ± 0.76 ^a^	5.79 ± 1.38 ^b^	4.65 ± 0.98 ^c^	4.05 ± 0.40 ^c^	3.85 ± 1.07 ^c^	3.69 ± 0.75 ^c^

^1^ Values are means ± standard deviation (SD); ^2^ Different letters differ from each other in the same type in different medium; ^3^ Repeated measures; one-way analysis of variance (ANOVA) of data.

**Table 2:** Cross-correlation matrix of variables measured in breast cancer cases under various culture media ^1,2^.

	B6	MNBN	NBUD	NPB	MONO	APO
MNBN	−0.764 ***	-				
NBUD	−0.563 ***	0.712 ***	-			
NPB	−0.615 ***	0.538 ***	0.426 **	-		
MONO	−0.448 ***	0.577 ***	0.440 **	0.234	-	
APO	−0.761 ***	0.739 ***	0.502 ***	0.356 *	0.216 *	-
NEC	−0.601 **	0.621 **	0.605 **	0.362 *	0.516 **	0.523 **

^1^ Values are estimated spearman correlation coefficients; ^2^ *** *p* < 0.001; ** *p* < 0.01; * *p* < 0.05.

**Table 3:** Cross-correlation matrix of variables measured in controls under various culture media ^1,2^.

	B6	MNBN	NBUD	NPB	MONO	APO
MNBN	−0.582 ***	-				
NBUD	−0.574 ***	0.701 ***	-			
NPB	−0.609 ***	0.620 ***	0.419 **	-		
MONO	−0.411 ***	0.472 ***	0.504 **	0.381 *	-	
APO	−0.663 ***	0.505 ***	0.528 ***	0.325 *	0.372 *	-
NEC	−0.510 ***	0.610 ***	0.592 ***	0.430 **	0.654 **	0.550 **

^1^ Values are estimated spearman correlation coefficients; ^2^ *** *p* < 0.001; ** *p* < 0.01; * *p* < 0.05.

**Table 4:** Effect of B6 and breast cancer status on GSACV ^1,2,3^.

Source of Variation	B6	Status	Interaction
	V%	V%	V%
MNBN	46.15 ***	5.12 *	0.88
NPB	30.13 **	4.14 *	0.30
NBUD	29.66 ***	0.48	0.30
MONO	3.14	0.04	1.56
APO	31.65 ***	0.61	1.87
NEC	40.15 ***	5.45 *	2.44

^1^ Values determined by two-way ANOVA; ^2^ *** *p* < 0.0001, ** *p* < 0.001, * *p* < 0.05; ^3^ Effect size is expressed as the ratio of the differences between the mean values for the two categories of each independent variable.

**Table 5:** Effect contribution to the strength and variation analysis of B6 and *SHMT C1420T* polymorphisms on GSACV by two-way ANOVA analysis.

GSACV	MNBN			MONO			NBUD			NPB			APO			NEC		
	F	*p*	% of Variation	F	*p*	% of Variation	F	*p*	% of Variation	F	*p*	% of Variation	F	*p*	% of Variation	F	*p*	% of Variation
**Controls**																		
Genotype (*SHMT*)	97.87	0.001 ***	21.10	6.36	0.002 **	2.18	0.383	0.682	0.17	2.54	0.081	1.47	0.764	0.467	0.43	4.161	0.017 *	2.31
B6	66.92	0.001 ***	36.07	48.82	0.001 ***	41.87	26.88	0.001 ***	30.42	14.41	0.001 ***	20.81	16.32	0.001 ***	22.73	14.30	0.001 ***	19.88
B6-genotype interaction	1.677	0.087	1.81	1.83	0.056	3.14	1.755	0.07	3.97	0.78	0.647	2.25	0.82	0.609	2.29	1.009	0.436	2.81
**Cases**																		
Genotype (*SHMT*)	25.31	0.001 ***	30.36	4.788	0.009 **	1.56	8.286	0.001 ***	3.21	1.690	0.187	1.03	1.322	0.266	0.83	2.10	0.125	1.17
B6	49.97	0.001 ***	16.58	26.51	0.001 ***	21.6	12.33	0.001 ***	11.94	4.720	0.001 ***	7.19	8.377	0.001 ***	13.09	4.071	0.002 **	5.68
B6-genotype interaction	1.116	0.35	0.74	1.012	0.434	1.65	2.014	0.033*	3.90	0.478	0.904	1.46	0.839	0.591	2.62	1.30	0.232	3.63

*** *p* < 0.001; ** *p* < 0.01; * *p* < 0.05.

**Table 6:** Effect contribution to the strength and variation analysis of B6 and *MS A2756G* polymorphisms on GSACV by two-way ANOVA.

GSACV	MNBN			MONO			NBUD			NPB			APO			NEC		
	F	*p*	% of Variation	F	*p*	% of Variation	F	*p*	% of Variation	F	*p*	% of Variation	F	*p*	% of Variation	F	*p*	% of Variation
Controls																		
Genotype (*MS*)	5.134	0.007 **	1.31	2.147	0.119	0.76	3.334	0.037 *	1.45	4.775	0.009 **	2.76	0.289	0.749	0.16	4.174	0.017 *	2.18
B6	44.52	0.001 ***	28.45	39.95	0.001 ***	35.3	16.31	0.001 ***	17.77	14.01	0.001 ***	20.23	5.942	0.001 ***	7.98	13.93	0.001 ***	18.19
B6-genotype interaction	3.465	0.001 ***	4.43	1.89	0.046 *	3.35	1.615	0.103	3.52	1.621	0.101	4.68	3.653	0.001 ***	9.81	1.957	0.039 *	5.11
Cases																		
Genotype (*MS*)	10.33	0.001 ***	2.89	8.801	0.001 ***	2.23	1.766	0.173	0.72	2.256	0.107	1.28	2.637	0.078	1.54	6.208	0.002 **	3.57
B6	29.62	0.001 ***	20.68	37.41	0.001 ***	23.69	19.57	0.001 ***	19.86	8.249	0.001 ***	11.68	4.779	0.001 ***	6.98	8.837	0.001 ***	12.71
B6-genotype interaction	4.164	0.001 ***	5.81	3.507	0.001 ***	4.44	2.275	0.003 **	5.58	1.011	0.435	2.86	2.565	0.006 **	7.49	0.648	0.772	1.86

*** *p* < 0.001; ** *p* < 0.01; * *p* < 0.05.

**Table 7:** Effect contribution to the strength and variation analysis of B6 and *MTRR A66G* polymorphisms on GSACV by two-way ANOVA analysis.

GSACV	MNBN			MONO			NBUD			NPB			APO			NEC		
	F	*p*	% of Variation	F	*p*	% of Variation	F	*p*	% of Variation	F	*p*	% of Variation	F	*p*	% of Variation	F	*p*	% of Variation
Controls																		
Genotype (*MTRR*)	0.626	0.536	0.19	0.383	0.682	0.13	0.221	0.801	0.10	2.229	0.11	1.33	1.117	0.329	0.64	0.265	0.877	0.04
B6	18.01	0.001 ***	13.79	13.75	0.001 ***	11.74	9.152	0.001 ***	10.03	11.38	0.001 ***	17.03	2.618	0.025 *	3.37	3.534	0.004 **	5.09
B6-genotype interaction	1.105	0.360	1.69	1.193	0.296	2.04	0.866	0.566	1.90	0.924	0.512	2.76	1.595	0.109	4.55	0.517	0.937	1.49
Cases																		
Genotype (*MTRR*)	2.625	0.075	0.84	5.499	0.005 **	1.40	3.36	0.036 *	1.44	0.501	0.606	0.29	2.879	0.058	1.72	0.335	0.716	0.19
B6	33.38	0.001 ***	26.69	51.12	0.001 ***	32.47	23.67	0.001 ***	25.31	8.116	0.001 ***	11.95	9.355	0.001 ***	13.95	12.31	0.001 ***	17.72
B6-genotype interaction	1.949	0.040 *	3.12	4.457	0.001 ***	5.66	0.919	0.516	1.97	0.610	0.805	1.80	1.845	0.054	5.50	1.502	0.139	4.32

*** *p* < 0.001; ** *p* < 0.01; * *p* < 0.05.

## Supplementary Materials

Supplementary materials can be found at http://www.mdpi.com/1422-0067/17/7/1003/s1.
