# Supplementary material for: The Role of Genetic Polymorphisms as Related to One-Carbon Metabolism, Vitamin B6, and Gene–Nutrient Interactions in Maintaining Genomic Stability and Cell Viability in Chinese Breast Cancer Patients
Source: Int J Mol Sci. 2016 Jun 24;17(7):1003. doi: 10.3390/ijms17071003 (PMC4964379; doi:10.3390/ijms17071003)
Supplement: Supplementary file 1 [file ijms-17-01003-s001.pdf]

Supplementary Materials: The Role of Genetic Polymorphisms as Related to One-Carbon Metabolism, Vitamin B6, and Gene-Nutrient Interactions in Maintaining Genomic Stability and Cell Viability (GSACV) in Chinese Breast Cancer Patients

Xiayu Wu, Weijiang Xu, Tao Zhou, Neng Cao, Juan Ni, Tianning Zou, Ziqing Liang, Xu Wang and Michael Fenech

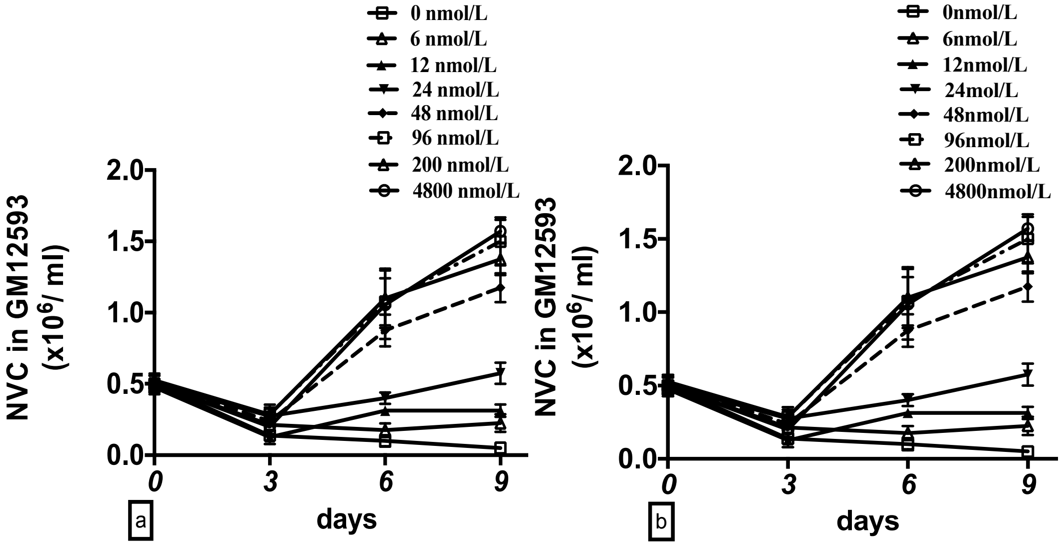

Figure S1. Growth of cell lines of GM12593 (**a**) and GM13705 (**b**) in medium containing various concentrations of B6 (values are the means ± SEM).
